# The Challenge of Coping with Dry Atmospheres: Structural and Physiological Adaptations to High VPD in Terrestrial Plants

**DOI:** 10.3390/plants15203216

**Published:** 2026-10-20

**Authors:** Eustaquio Gil-Pelegrín, Juan Pedro Ferrio, Antonio Diaz-Espejo, Victoria Fernández, Luna Morcillo, Domingo Sancho-Knapik, Alberto Vilagrosa, José Javier Peguero-Pina

**Affiliations:** 1 Estación Experimental Aula Dei (EEAD-CSIC), 50059 Zaragoza, Spain; jpferrio@eead.csic.es; 2 Plant Ecophysiology and Irrigation (ECOVER) Group, Instituto de Recursos Naturales y Agrobiologia de Sevilla (IRNAS-CSIC), 41012 Sevilla, Spain; a.diaz@csic.es; 3 Department of Systems and Natural Resources, School of Forest Engineering, Universidad Politécnica de Madrid, 28040 Madrid, Spain; v.fernandez@upm.es; 4 Fundación Centro de Estudios Ambientales del Mediterráneo (CEAM), Joint Research Unit University of Alicante—CEAM, 03690 Alicante, Spain; luna@ua.es (L.M.); a.vilagrosa@ua.es (A.V.); 5 Centro de Investigación y Tecnología Agroalimentaria de Aragón (CITA), 50059 Zaragoza, Spain; dsancho@cita-aragon.es (D.S.-K.); jjpeguero@cita-aragon.es (J.J.P.-P.); 6 Instituto Agroalimentario de Aragón-IA2-(CITA-Universidad de Zaragoza), 50013 Zaragoza, Spain; 7 Unidad de Suelos y Riegos, CITA-EEAD-CSIC, 50059 Zaragoza, Spain; 8 Department of Ecology, University of Alicante, 03690 Alicante, Spain

**Keywords:** atmospheric dryness, cuticle, plant hydraulics, stomata, vapor pressure deficit, water saver, water spender

## Abstract

Terrestrial plants have developed structural and physiological traits to maintain water balance under variable environments. Protective surfaces reduce water loss but also restrict CO_2_ diffusion. This constraint has been overcome via stomata, which control gas exchange between plant and atmosphere. Moreover, the development of an efficient xylem allowed long-distance water transport and higher growth. The soil–plant–atmosphere continuum (SPAC) establishes that water fluxes are governed by water potential gradients and are modulated by plant hydraulic traits. The atmosphere is the ultimate driver of water fluxes. It determines the strength of the gradient that sustains transpiration, establishing a strong vapor pressure gradient between the leaf and the surrounding air (i.e., the vapor pressure deficit, VPD). We focus on how high VPD by itself can negatively affect plant functioning. Specifically, we analyze: (i) cuticle as a protective surface to reduce water loss, (ii) stomata as the regulator of gas exchange between plant and atmosphere, (iii) plant water transport at long distances to supply water to transpiring surfaces, and (iv) drought-avoidance strategies reflecting contrasting coordination between stomatal regulation and hydraulic supply. VPD is a central factor shaping plant structure, physiology and survival, revealing the need for a coordinated response at whole-plant level.

## 1. Introduction

The colonization of terrestrial environments by plants represented a major evolutionary challenge, as the transition from an aquatic to an aerial habitat exposed organisms to a completely new set of abiotic stresses [1]. Among these, the risk of desiccation was particularly critical, given the absence of a continuous water medium to sustain hydration [2]. In addition, early land plants faced increased temperature fluctuations, higher levels of ultraviolet radiation, and mechanical stresses associated with exposure to air [1]. These constraints required the evolution of novel structural and physiological traits to maintain water balance, protect cellular integrity, and ensure survival under highly variable environmental conditions. In this regard, Weatherley [3] articulated the mechanisms that plants had to develop to colonize the terrestrial environment. Thus, an organism composed entirely of living cells would either have to remain flattened, keeping all tissues near a water source and thus limiting vertical growth, or develop a protective surface to reduce water loss. While the former constrains structural complexity, the latter introduces a critical trade-off, as a highly impermeable barrier would also restrict carbon dioxide diffusion, thereby impairing photosynthesis.

Terrestrial plants resolved this constraint not through a perfectly selective surface, but by combining a largely water-resistant epidermis with specialized microscopic pores—stomata—that enable the control of gas exchange between plant and atmosphere. Moreover, in addition to adequate waterproofing, Weatherley [3] also considered that the evolution of an efficient xylem transport system enabled plants to grow large on land by allowing long-distance water movement, thereby overcoming the low permeability of individual cells. On the other hand, considering current knowledge on xylem function under high water tension, this condition of efficiency should also be accompanied by that of safety. Consequently, terrestrialization was not a single event but rather a complex and gradual process involving multiple adaptive innovations that enabled plants to persist outside aquatic environments.

**Figure 1:**
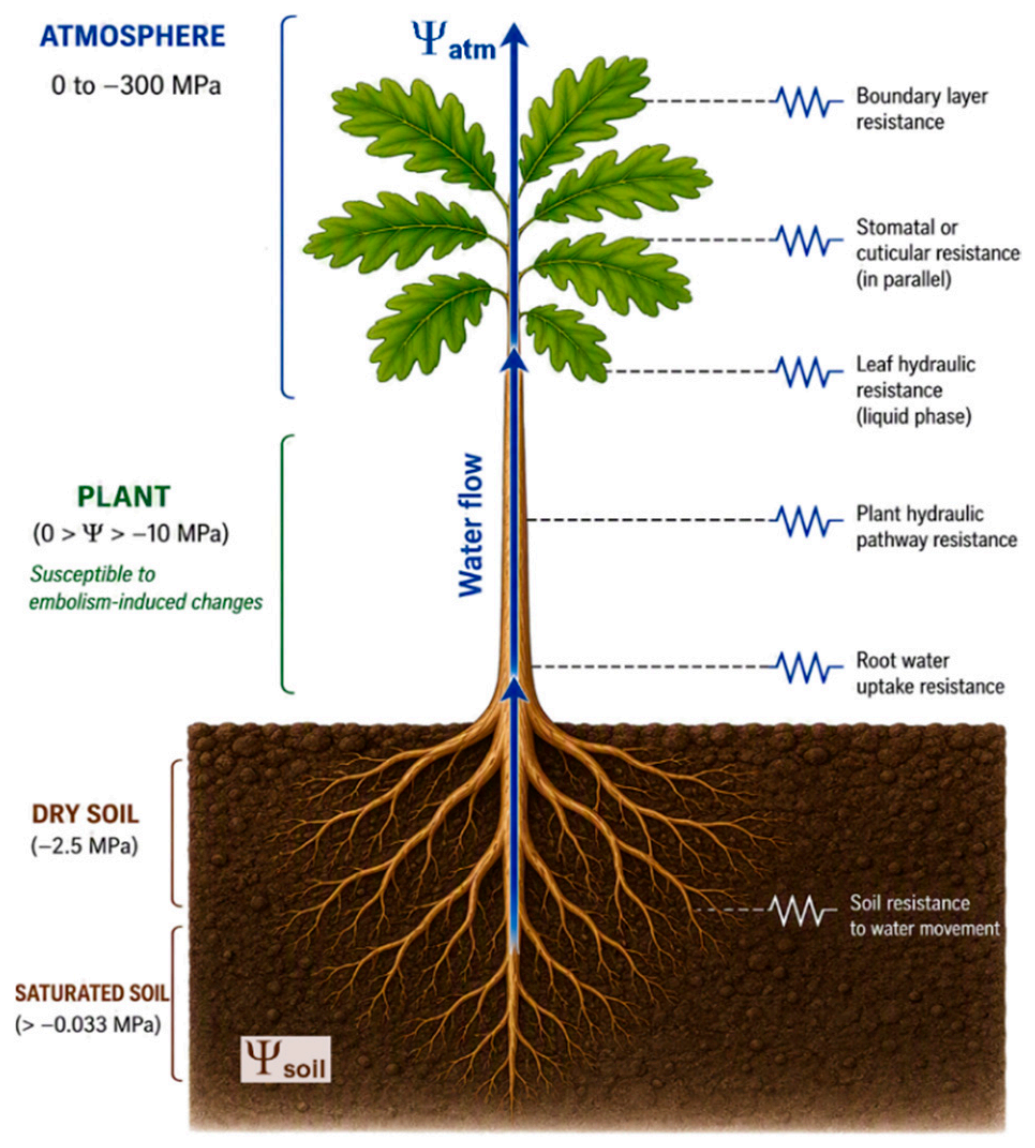
**Conceptual frame of the Soil–Plant–Atmosphere Continuum (SPAC).** Water flows along a gradient of decreasing water potential from the soil (Ψ_soil_) to the atmosphere (Ψ_atm_). The water pathway comprises serial resistances along the liquid phase (soil water movement, root water uptake, xylem transport, leaf hydraulic resistance) and the gas phase (stomatal or cuticular resistances, and the atmospheric boundary layer). Plant water status is maintained within a finite physiological range but may be altered by embolism-induced reductions in hydraulic conductivity, resulting in impaired water transport and increased hydraulic dysfunction risk.

The challenge of living surrounded by the atmosphere, a medium which can reach extremely negative water potentials (Ψ), defines most of the adaptations of plants that have been extensively studied by plant physiologists. The movement of water through the soil–plant–atmosphere system has been classically interpreted as a “continuum”, driven by water potential gradients from a main source (soil water) to a sink (free air), through a pathway regulated by a series of resistances, as proposed by van den Honert [4] (Figure 1).

**Figure 2:**
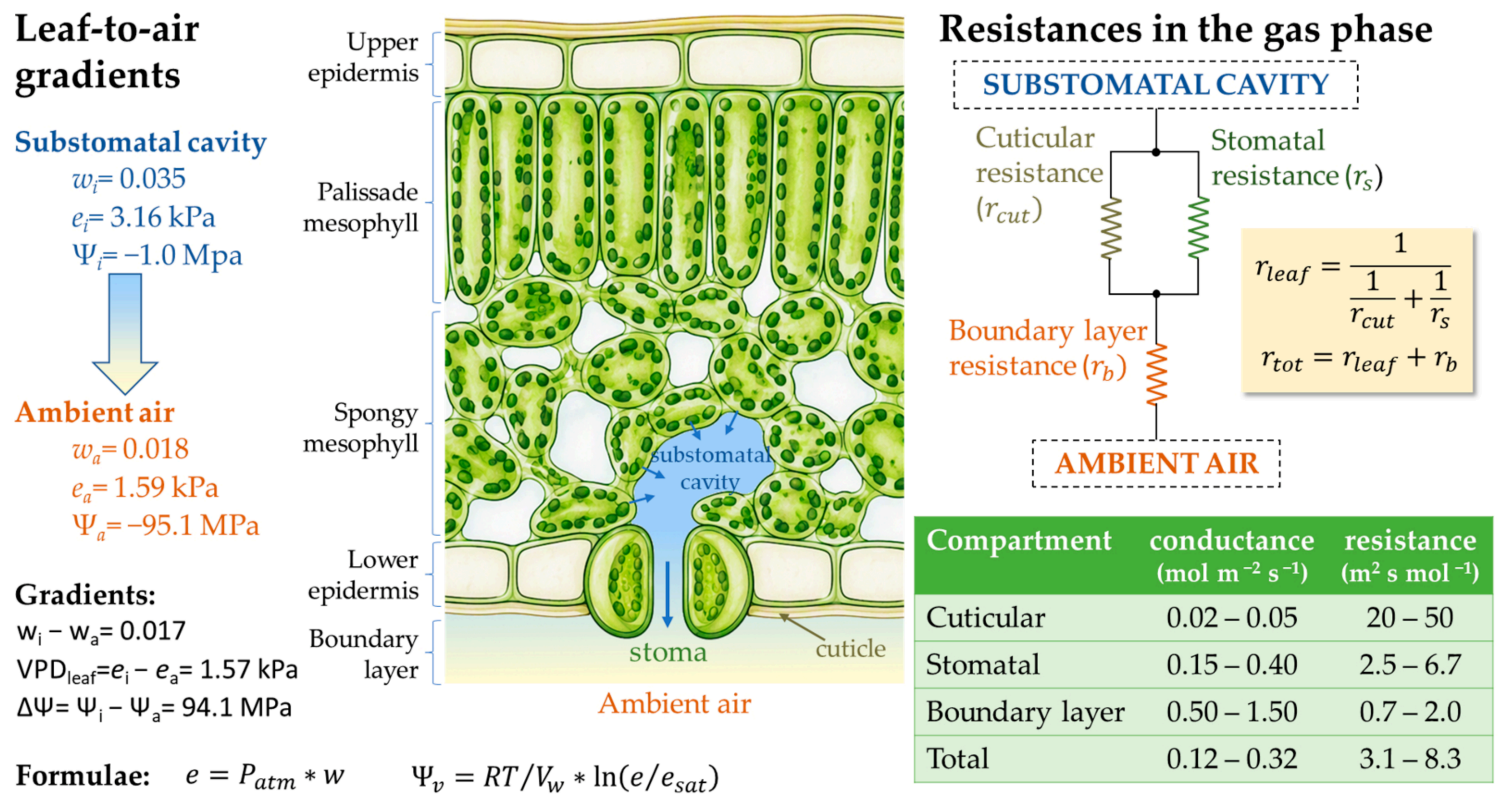
**Main gradients and resistances along the gas phase.** To the left, values for molar ratios inside the leaf (*w_i_*) and in the atmosphere (*w_a_*), and the leaf-to-air molar gradient (*w_i_ − w_air_*), and their equivalence in terms of vapor pressure (*e_i_*, *e_a_*, *e_i_ − e_a_*) and water potential (*Ψ*_i_, *Ψ*_a_, *Ψ*_i_ − *Ψ*_a_). The central part of the figure depicts a schematic leaf section with the most relevant compartments dealt with in this review. To the right, electrical analogy of the key resistances in the gas phase, their contribution to total leaf conductance (*g_tot_* = 1/*r_tot_*), and representative values for non-stressed, sun-exposed leaves. The formulae show the conversion between different magnitudes [5], where *P*_atm_ is atmospheric pressure, *R* is the universal gas constant, *T* is air temperature, *V_w_* is the molar volume of liquid water and *e_sat_* is saturation vapor pressure (note that *e*/*e_sat_* is relative humidity—RH—shown as a decimal fraction). Calculations were made assuming the same leaf and air temperature (25 °C), ambient RH = 50%, and vapor pressure inside the leaf in equilibrium with leaf water potential (*Ψ_leaf_* = −1 MPa), therefore departing slightly from saturation (leaf RH = 99.3%).

Air water potential describes atmospheric dryness in thermodynamic terms and, as in the liquid phase, the leaf-to-air water potential gradient determines the direction of water movement. However, in the gas phase the magnitude of flux is governed by the gradient in water vapor concentration, following Fick’s law, and hence the water-vapor mole-fraction gradient is the driving force determining transpiration rates (*E*) [5]:

*E* = *g_tot_* × (*w_i_* − *w_a_*) (1)

where *w_i_* and *w_a_* stand for the molar ratio of water vapor inside the leaf and in the atmosphere, respectively, and *g_tot_* is the total conductance to water vapor from the substomatal cavity to the ambient air (see Figure 2).

Since the intercellular airspaces of the leaf are typically close to saturation, while the surrounding atmosphere is usually subsaturated, a strong vapor pressure gradient is established between the internal part of the leaf and the surrounding air. This gradient is quantified as the leaf-to air vapor pressure deficit (VPD), defined as the difference between saturation vapor pressure at leaf temperature and the actual vapor pressure of the air. Although the molar gradient is the direct physical driver of transpiration, VPD is a convenient and more easily measurable proxy, whose limitations are usually small within a similar pressure range (for the implications of using VPD instead of the molar gradient see Appendix A). Whereas VPD can be directly determined by measuring temperature and humidity, the molar gradient requires accurate estimates of total atmospheric pressure, which are often absent or incompletely reported in ecological studies. On the other hand, most scientific reports describe plant responses as a function of VPD [6,7,8]; therefore, we will focus here on the response to VPD, as the most widely used proxy of atmospheric dryness and the driving force for transpiration.

The aim of the present review is to present current knowledge and future directions on how high VPD values can negatively affect plant survival, even when soil water availability is not a limiting factor. Specifically, in this review we will discuss those previous experimental designs that consider the effect of atmospheric dryness itself, with a clear separation from soil water availability. To do this, we will follow the postulates of Weatherley [3], analyzing the following aspects in the context of a high evaporative demand: (i) cuticle as a protective surface to reduce water loss, (ii) stomata as the structure devoted to regulating gas exchange (CO_2_ and H_2_O) between plant and atmosphere, (iii) water transport at long distances within the plant to supply water to transpiring surfaces, and (iv) drought-avoidance strategies reflecting contrasting coordination between stomatal regulation and hydraulic supply.

## 2. Role of Cuticle as a Protective Surface to Reduce Water Loss

The cuticle has been proposed as one of the most critical innovations enabling land colonization and plant survival in terrestrial habitats [2]. The cuticle, which covers the outer cell wall of aerial organs, plays a fundamental role in reducing uncontrolled water loss and protecting tissues from environmental stress factors [9]. Its ubiquity across land plants further supports its importance as a key adaptive feature associated with terrestrial life, highlighting its central role in enabling plants to overcome the physiological constraints imposed by a non-aquatic habitat [10].

The evolution of the plant cuticle is intimately linked to the transition of plants from aquatic to terrestrial environments. During this transition, early embryophytes encountered severe selective pressures, particularly water loss, ultraviolet radiation, and thermal variability, which required the development of effective protective barriers [1,2]. Comparative genomic and phylogenetic analyses indicate that the capacity to synthesize cuticle components, such as cutin and waxes, originated in the last common ancestor of land plants [11]. This suggests that cuticle was an early and fundamental innovation associated with terrestrialization that was progressively modified as plants diversified [12,13]. In early-diverging lineages such as bryophytes, the cuticle is generally thinner and chemically simpler than in vascular plants, yet it already performs essential protective roles against desiccation and environmental stress [14,15]. As plant lineages diversified, the composition and structural complexity of the cuticle increased, with higher proportions of cutin, very-long-chain waxes, and specialized lipid derivatives enhancing its barrier properties [11]. Concurrently, regulatory networks controlling cuticle biosynthesis became more elaborate, as evidenced by the conservation of key transcription factors involved in cutin production across land plants [16]. Additionally, early cuticles may have been enriched in phenolic compounds, representing an ancestral state that contributed to mechanical reinforcement and stress tolerance before the evolution of more complex polymers such as lignin [17]. Overall, the evolutionary trajectory of the cuticle reflects a gradual increase in structural sophistication and functional efficiency, underpinning the successful radiation of plants across terrestrial ecosystems and highlighting its central role as a dynamic interface between plants and their environment. The cuticle is present in aerial plant surfaces such as leaves, stems, flowers, and fruits [18], although recent findings have demonstrated the existence of a cuticle-like layer in the root cap of *Arabidopsis thaliana*, suggesting that this structure may not be exclusively restricted to aerial organs [19]. The protective properties of the cuticle are closely associated with its structural organization and chemical composition at micro- and nanoscale levels, although establishing clear relationships between these factors remains challenging due to methodological limitations [20].

Chemically, the cuticle is predominantly composed of lipid-derived materials, including cuticular waxes, cutin, and sometimes cutan, along with minor amounts of phenolic compounds and mineral elements [21,22,23]. Waxes can be located either on the cuticle surface (epicuticular) or embedded within the cuticular matrix (intracuticular) [24]. However, the relative contribution of these wax fractions to water barrier properties remains a matter of debate [25,26]. Cutin, a structural polyester synthesized by epidermal cells [22], is mainly composed of C_16_ and C_18_ hydroxy fatty acid monomers [2,27]. By contrast, cutan is an alternative insoluble and non-saponifiable polymer [21,28], and its classification has recently been refined based on degradability criteria [29]. In addition, phenolic compounds have been shown to contribute to the structural, mechanical, and functional properties of the cuticle [30]. Moreover, recent studies have also reported the presence of polysaccharides within the cuticle of leaves and fruits [22,23,31]. This evidence supports a revised interpretation of the cuticle as a specialized and lipid-rich region of the primary cell wall [20], analogous in some respects to lignified or suberized cell walls [32].

Elucidating the relationship between cuticle composition and structure, including potential links between chemical components, remains difficult to evaluate due to its composite nature [33]. The cuticle comprises both hydrophobic components, such as waxes and cutin, and hydrophilic components, primarily polysaccharides, arranged in a heterogeneous manner at different spatial scales [20]. Ultrastructural studies revealed diverse structural patterns [18], but interpretation in the absence of specific techniques such as immuno-labelling [22,31] or selective chemical removal [34] is difficult owing to the hundreds of chemical compounds forming the cuticle. Furthermore, comparison of cuticles from different studies is often complicated as they can substantially vary according to the sample preparation methods applied, including tissue fixation, staining, embedding and post fixation [35] with the potential occurrence of artifacts [36].

**Figure 3:**
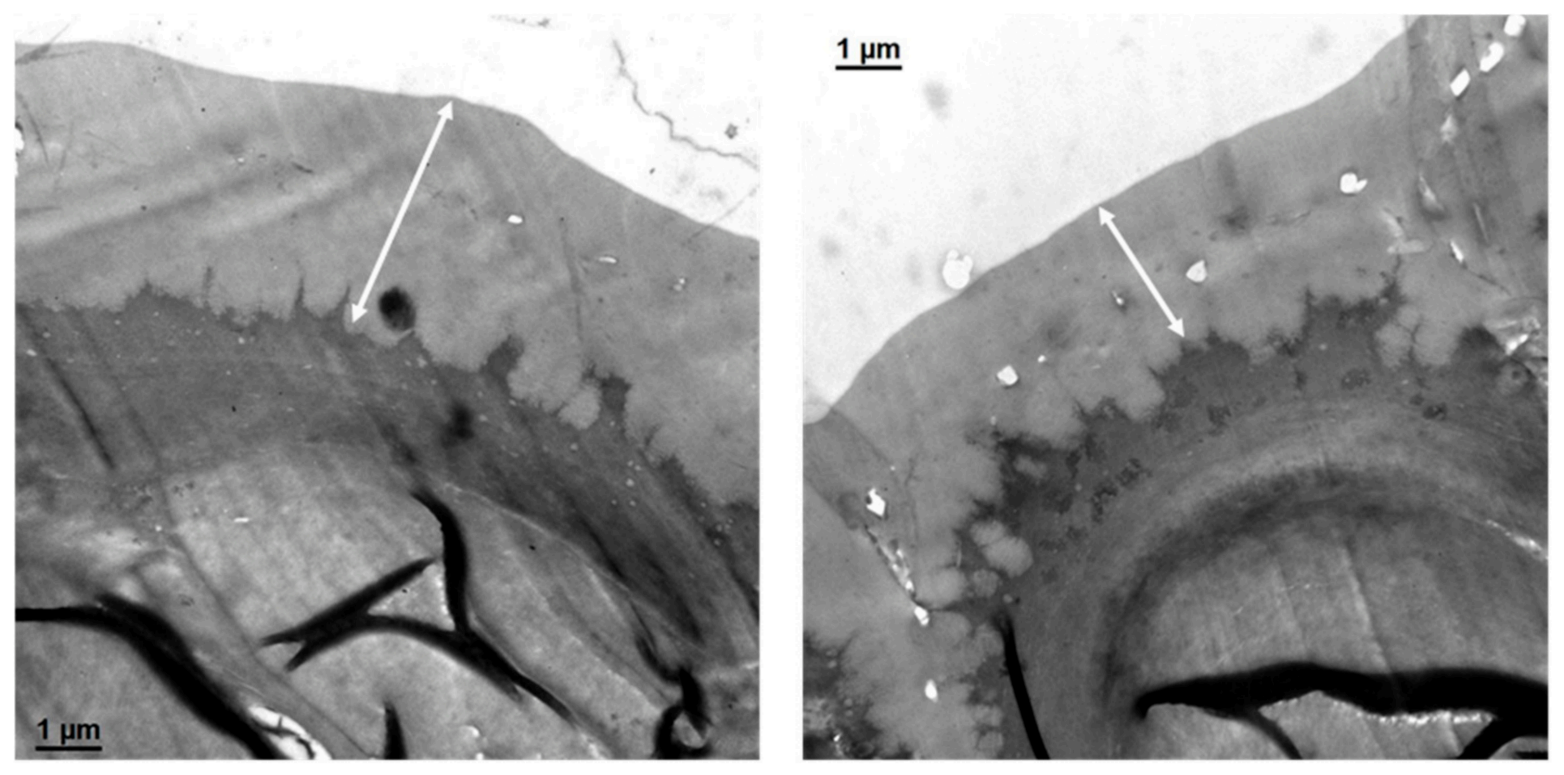
Transmission electron micrographs of the cuticle and outer cell wall for needles of *Pinus uncinata* Ram. grown at lower (**left image**) and higher (**right image**) elevation in the Pyrenees. The white arrows represent cuticle thickness.

From an ecophysiological perspective, the cuticle is essential for regulating residual water loss through the epidermis when stomata are fully closed, referred to as cuticular transpiration or minimum conductance (*g*_min_). This is particularly relevant in alpine and treeline ecosystems, where environmental conditions impose severe constraints on plant water balance. In these habitats, plants are subjected to short growing seasons, low soil temperatures, and high evaporative demand, which may result in “frost drought” conditions [37]. Under such circumstances, stomata are largely closed, and minimizing cuticular water loss becomes critical for maintaining tissue hydration [38]. In this regard, a reduced growing season length at higher elevations may limit cuticle maturation (i.e., cuticle thickness), leading to increased *g*_min_ and, consequently, a higher risk of suffering frost drought during winter [39] (Figure 3). By contrast, Anfodillo et al. [38] reported the existence of thicker cuticles at higher elevations without corresponding changes in *g*_min_. These contrasting results indicate that the relationship between cuticle structure, composition, and function remains complex and is affected by multiple factors, such as plant species, growing habitat or organ ontogeny.

*g*_min_ is also critically important in seasonally dry and arid environments. Once stomatal regulation can no longer substantially reduce water loss, *g*_min_ determines the residual transpiration rate and therefore controls how rapidly the remaining water stored in leaves, stems, and other living tissues is depleted. Consequently, even relatively small differences in *g*_min_ can produce large differences in the time required for a plant to reach critical thresholds of tissue dehydration and hydraulic dysfunction. Mechanistic models of plant desiccation identify *g*_min_, together with plant hydraulic capacitance, accessible water pools, xylem vulnerability, and evaporative demand, as a major determinant of the time to hydraulic failure and mortality after stomatal closure [40,41,42]. Importantly, *g*_min_ should not be treated as a constant species-specific parameter. *g*_min_ generally increases with leaf temperature, as thermal changes in cuticular waxes and other lipid domains increase membrane permeability and compromise the effectiveness of the transpiration barrier [9,41,42,43]. This temperature dependence creates potentially dangerous positive feedback under hot and dry conditions: stomatal closure restricts evaporative cooling, leaf temperature rises, cuticular conductance increases, and stored water is depleted more rapidly. Thus, the thermal stability of the cuticular barrier, rather than *g*_min_ measured at a single standard temperature, is likely to be a key functional trait determining plant survival during combined heat and atmospheric dryness.

**Figure 4:**
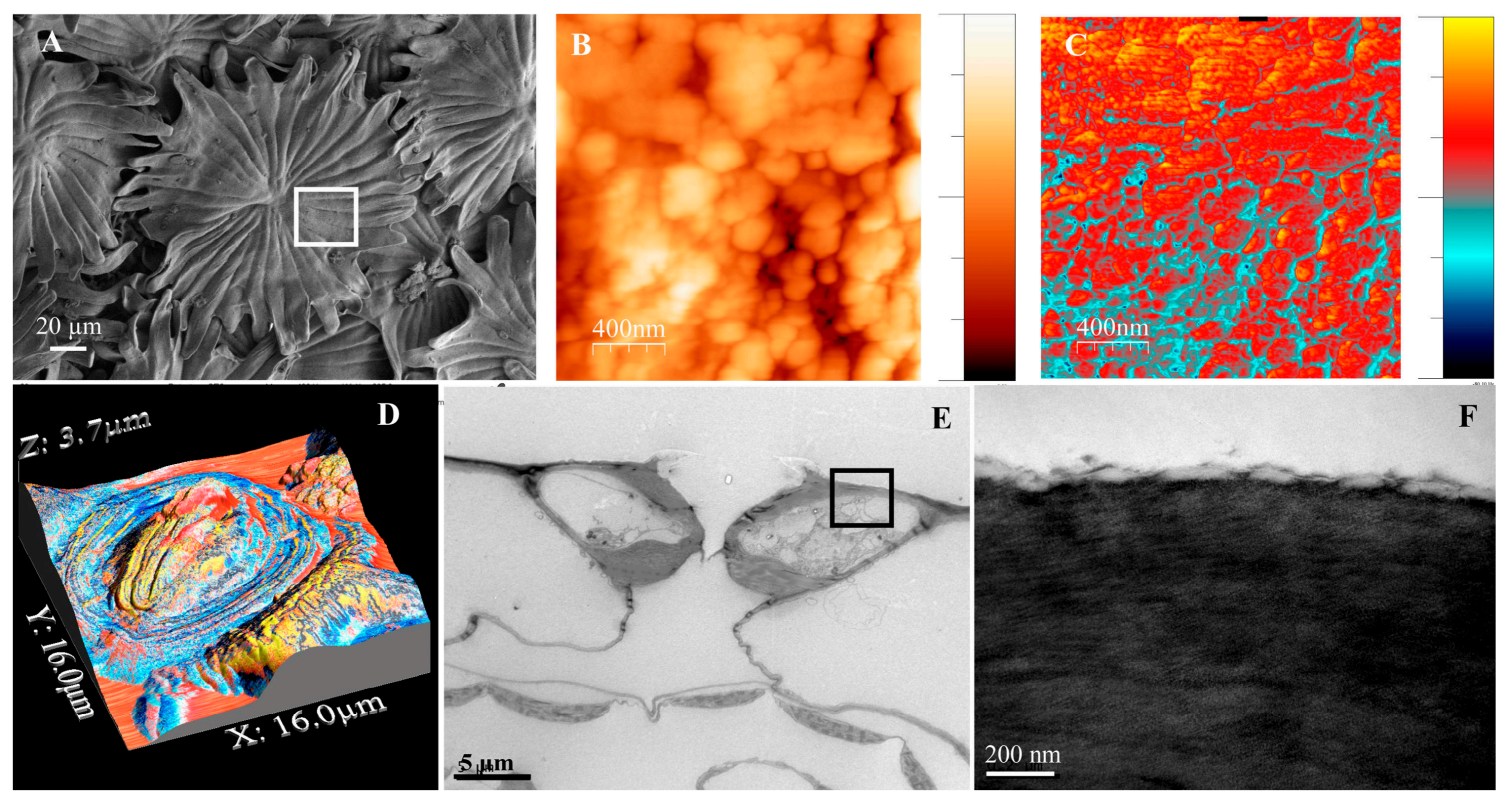
Electron microscopy (**A**,**E**,**F**) and AFM (**B**–**D**) micrographs of olive leaf trichomes (**A**–**C**) and lettuce leaf stomata (**D**–**F**). Trichomes covering the abaxial surface of olive leaves (**A**), with a close up showing the topography (**B**) and wettability (**C**); maps of a trichome area. Images of a lettuce leaf stoma showing its surface wettability by AFM (**D**), TEM cross-section (**E**) and a discontinuous cuticle area of the guard cell (**F**). AFM heat maps of surface wettability (**C**,**D**) show the occurrence of hydrophilic (blue) and hydrophobic (yellow to red) material.

Current investigations analyzing cuticle surfaces at the nanoscale are providing evidence for the chemical heterogeneity of some cuticle areas in model epidermal cells and plant organs [44,45,46] (see Figure 4). As noted above, cuticular ultrastructure may vary when comparing the same organ of different species, but different patterns have been reported between leaf epidermal cells for olive trichomes and lettuce guard cells compared to pavement cells [45,46]. Regarding wax composition, Hegebarth et al. [47] recorded changes between trichomes and pavement cells of *Arabidopsis thaliana* leaves. A high frequency of hydrophilic areas at the surface of lettuce leaf guard cells has been determined by Atomic Force Microscopy (AFM), and this can also be observed in Transmission Electron Microscopy (TEM) cross-sections, where cuticle interruptions are identified (Figure 4F). The direct exposure of cell wall material to the atmosphere (Figure 4) may have multiple functional implications for the transport of gases and liquids (e.g., peristomatal transpiration, as described below), surface interactions or surface mechanical properties which should be elucidated in future investigations.

Finally, from an evolutionary perspective, it should be noted that some spermatophytes that have evolved towards occupying freshwater habitats show a secondary reduction in cuticle development associated with the transition back to aquatic environments. In this sense, *Potamogeton octandrus*, a heterophyllous aquatic angiosperm capable of producing both submerged and floating leaves, exhibits pronounced anatomical plasticity in response to contrasting environmental conditions [48]. Thus, submerged leaves of *P. octandrus* exhibit a reduced or absent cuticle, with a smooth epidermal surface devoid of waxy ornamentation. In contrast, floating leaves, which are exposed to the atmosphere, display a thicker epidermis, the presence of stomata, and a well-developed cuticular wax layer, reflecting the need to regulate transpiration and gas exchange in air. In this species, cuticle development is suppressed in the aquatic environment to facilitate direct exchange with the surrounding water, while being retained and reinforced in aerial tissues to prevent dehydration. Indirectly, this process reinforces the idea that the cuticle is a structure linked to survival in an aerial environment.

Overall, the plant cuticle represents a structurally and chemically diverse interface that has played a fundamental role in plant adaptation to terrestrial environments as a protective surface to reduce water loss. Despite significant advances, its functional characterization remains incomplete, particularly regarding the interplay between composition, ultrastructure, environmental responses, and outward water-vapor flux.

## 3. Stomatal Response to Atmospheric Water Deficit

The evolution of a continuous, lipid-rich cuticle was essential for terrestrial life because it restricted uncontrolled water loss, but the same diffusion barrier limited CO_2_ entry into photosynthetic tissues. Stomata can therefore be interpreted as regulated discontinuities in the cuticle that reconcile carbon acquisition with the prevention of excessive dehydration [49,50,51,52]. Plants have acquired the ability to modify their stomatal apertures in response to pathogens and environmental conditions [53]. This trade-off becomes particularly acute at high VPD, when opening the pathway for CO_2_ simultaneously exposes the moist internal leaf to a steep gradient driving water vapor towards the atmosphere. The mechanistic question is thus not simply whether stomata “sense VPD”, but how atmospheric evaporative demand perturbs leaf water balance and how that perturbation is converted into stomatal movement [7,54,55].

The stomatal apparatus is not an isolated pair of guard cells. Aperture emerges from the hydraulic and mechanical integration of guard cells, adjacent epidermal or subsidiary cells, the substomatal cavity, mesophyll tissues, and the xylem pathway supplying them [56]. This tissue-level perspective provides the basis for evaluating the classical peristomatal-transpiration hypothesis, modern hydroactive models, and their evolutionary variation across land plants.

### 3.1. From Atmospheric Evaporative Demand to Stomatal Movement

Transpiration is determined by the vapor gradient between internal evaporating surfaces and the atmosphere, together with stomatal, boundary-layer, and cuticular conductances. An increase in VPD therefore tends initially to increase water loss. At hydraulic steady state, the water potential of a tissue can be expressed schematically as:

*Ψ_j_ = Ψ_source_ − R_source−j_ E* (2)

where *Ψ_j_* is water potential at a given location, *Ψ_source_* is source water potential, *R_source−j_* is the effective hydraulic resistance between source and tissue, and *E* is transpiration. Tissue water status can consequently change through variation in source water potential, hydraulic resistance, or evaporative flux. VPD acts primarily through *E*, although its effects may be amplified if leaf or whole-plant hydraulic conductance declines during dehydration [54,56].

Stomatal aperture is positively related to guard-cell turgor but negatively related to the turgor of surrounding epidermal or subsidiary cells. Pressure-probe experiments show that the epidermis has a mechanical advantage over guard cells in several angiosperms [57]. If guard-cell and epidermal turgor decline similarly, the release of epidermal back-pressure may initially open rather than close to the pore. This transient hydropassive opening, termed the wrong-way response, is followed by a slower right-way closure that requires a disproportionate decline in guard-cell turgor, generally produced by active loss of guard-cell osmolytes [55,58].

The metabolically mediated adjustment of guard-cell osmotic content in response to leaf water status is described as hydroactive feedback. Increased VPD changes transpiration and local hydration; declining water status at a sensory site then activates ion and solute efflux from guard cells, reduces their turgor, and closes the pore. The sensed variable may be water potential, cell volume, relative water content, membrane tension, or an associated signal. ABA accumulation during leaf dehydration correlates more consistently with declining cell volume than with a universal turgor threshold, and guard cells, vascular tissues, phloem companion cells, and mesophyll have all been proposed as sensory or ABA-producing sites [59,60,61,62]. Any proposed VPD-response mechanism must therefore explain epidermal mechanics, wrong-way and right-way responses, and the similar effects of perturbing hydraulic demand and supply.

### 3.2. Peristomatal Transpiration: From a Local Humidity-Sensing Hypothesis to a Component of Integrated Leaf Hydraulics

The peristomatal-transpiration hypothesis establishes that dry air could induce stomatal closure with small changes in bulk leaf water status. Pioneer experiments proposed that guard cells or adjacent tissues experienced preferential water loss and acted as local humidity-sensitive elements [63,64,65]. Evaporation from external or internal guard-cell surfaces generated a larger decline in guard-cell water potential and turgor than in neighboring epidermal cells, causing closure without a specialized molecular humidity receptor or a substantial change in bulk leaf water potential [66,67].

The term includes at least three distinct processes: water loss through the external guard-cell cuticle; evaporation from internal guard-cell or adjacent peristomatal walls into the pore or substomatal cavity; and vapor exchange between guard cells and pore-channel air. These alternatives differ in evaporating surface, hydraulic supply pathway, resistance between guard and epidermal cells, and predicted response to altered water supply [54,55]. Indirect evidence suggest that permeability is heterogeneous around stomata and that aqueous compounds can use polar pathways associated with guard cells, stomatal ledges, trichomes, or cuticular discontinuities [68,69,70]. However, it is not demonstrated the existence of a quantitatively important outward water-vapor flux because phase, driving force, hydration state, and swelling of polar domains differ.

The classical hypothesis faces three main mechanistic constraints. First, preferential guard-cell evaporation must produce enough turgor loss to overcome epidermal mechanical advantage. This requires both appreciable local evaporation and a large effective hydraulic resistance between guard cells and neighboring epidermal tissues. Such sustained isolation is difficult to reconcile with their anatomical proximity, guard-cell aquaporins, substantial membrane water permeability, and rapid osmotic volume responses [71,72]. Second, the frequent wrong-way response constrains the magnitude and timing of preferential guard-cell drawdown. If external guard-cell evaporation immediately dominated local water relations, stomata should close without the transient opening produced by loss of epidermal back-pressure [73]. Third, stomata show similar steady-state responses when VPD increases, source water potential decreases, or hydraulic resistance increases, and these supply- and demand-side perturbations can yield comparable wrong-way/right-way sequences [74,75,76]. Pore-localized evaporation alone does not readily explain this symmetry, whereas hydroactive feedback to tissue water status does because both supply and demand alter hydration at the sensory site.

Altered-gas-diffusivity experiments provided an important test of direct humidity sensing. Replacing nitrogen in the air with helium (“helox” gas mixture) increases water-vapor diffusivity and partly separates ambient humidity from transpiration. Stomatal behaviour in helox was more closely associated with leaf water loss than with relative humidity itself, challenging the idea that stomata read external humidity as an independent variable [77]. Nevertheless, gas substitution also changes heat transfer and boundary-layer properties, and the foundational result has been replicated in relatively few taxa. Accurate leaf-temperature measurement is essential because small temperature errors strongly affect calculated leaf-to-air water vapor gradients [55]. Thus, the helox evidence supports a water-loss-mediated response but does not exclude subsidiary pore-scale effects.

Modern hydromechanical models can incorporate local water loss without assigning it the role of a universal sensor. Total water loss can be partitioned schematically as:

*E_total_ = E_stomatal_ + E_cuticular_ + E_peristomatal_* (3)

and guard-cell water potential as *Ψ_g_ = Ψ_supply_* − *R_g_E_g_*, where *E_g_* is the local flux supported by guard cells and *R_g_* its effective supply resistance. The key question is whether *R_g_E_g_* is large enough to generate a meaningful decline in guard-cell water potential relative to the epidermis. The Buckley et al. framework [58] explicitly couples guard-cell and epidermal turgor, epidermal mechanical advantage, hydraulic supply, evaporative demand, and active osmotic regulation. In this framework, local drawdown may contribute to guard-cell dehydration, but hydroactive regulation is needed to explain the full response to both demand- and supply-side perturbations [54,58].

Peristomatal water loss may nevertheless modify the amplitude and/or kinetics of hydroactive feedback. Local evaporation could intensify dehydration of guard cells, epidermis, or nearby mesophyll and thereby alter the signal initiating active closure. Its importance will depend on where water status is sensed. If sensing occurs in guard cells, the effect could be direct; if it occurs mainly in mesophyll or vascular tissues, external guard-cell evaporation would have less influence on ABA production. However, no study has yet demonstrated a causal sequence from preferential peristomatal evaporation to local ABA synthesis, and ABA-independent active processes also remain possible [59,78,79,80].

A related vapor-phase model proposes that guard cells are weakly coupled to liquid water in adjacent tissues, but are strongly coupled to humidity in the pore channel [81]. This can explain local humidity responses and feedforward-like behaviour, but requires a marked asymmetry in water exchange that remains uncertain. However, heat and mass-transfer models further show that evaporation is distributed among mesophyll, bundle-sheath, and epidermal surfaces and changes with anatomy, radiation, and temperature gradients [82,83].

The classical peristomatal hypothesis is therefore insufficient as a general autonomous explanation of VPD-induced closure, but local water exchange around stomata should not be dismissed. It may contribute to local water-potential gradients, modulate hydropassive and hydroactive kinetics, and generate spatial variation among stomata. Newly observed heterogeneity in guard-cell cuticle structure and wettability provides a strong rationale for renewed tests. However, functional significance requires direct or model-constrained estimates of local vapor conductance, guard-cell and epidermal water status, and response kinetics under independent manipulations of VPD, temperature, gas diffusivity, and hydraulic supply.

### 3.3. Evolutionary Variation in Hydropassive and Hydroactive Regulation

Comparative studies indicate that the relative importance of passive hydraulics and active biochemical regulation differs among land-plant lineages. In many ferns and lycophytes, short-term responses to increasing VPD or leaf excision are dominated by passive changes in guard-cell water relations, and the wrong-way response is absent or weak, consistent with limited epidermal mechanical advantage [84,85]. Exogenous ABA can close stomata in some taxa of these lineages, but responses are often weaker or slower, vary among species, or require high concentrations. Endogenous ABA appears less consistently involved in rapid atmospheric-dryness responses than in angiosperms [86,87,88,89].

In angiosperms, reduced humidity can induce expression of ABA-biosynthetic enzymes and increase leaf ABA within minutes. ABA promotes anion and K^+^ efflux, lowers guard-cell osmotic content, and provides a mechanism capable of overcoming epidermal back-pressure [59,78,79]. The response is not necessarily universal or exclusively ABA-dependent. Thus, several ABA-deficient or signaling mutants retain substantial VPD responses, suggesting redundancy and a role of ABA in both basal conductance and response gain [55,80]. Gymnosperms occupy a diverse intermediate position, with functional but generally slower ABA-mediated regulation that can be represented by integrated hydraulic–hormonal models [90]. These patterns are better interpreted as differences in speed, magnitude, and integration than as the absolute presence or absence of active control.

Pichaco et al. [91] recently proposed that rapid ABA-dependent hydroactive regulation was a key innovation contributing to angiosperm ecological dominance. Thus, dense venation and small, numerous stomata enhanced photosynthetic capacity. As a negative consequence, it also increased the hydraulic exposure and epidermis-guard-cell mechanical coupling, including the risk of wrong-way responses. Rapid hydroactive closure acted as a safeguard by reducing guard-cell turgor when water status declined and improving coordination among carbon gain, water loss, and hydraulic safety. The proposal should be regarded as an evolutionary hypothesis rather than a completed demonstration. It does not imply that responses to light, CO_2_, or metabolism are mediated by the same ABA pathway. Instead, hydroactive water-status control is viewed as an additional module within a broader stomatal signaling network [91].

### 3.4. Leaf Turgor, Hydraulic Conductance, and Active Stomatal Closure

The VPD response depends on the leaf capacity to maintain tissue hydration. Hydraulic capacitance can transiently buffer declining water potential by releasing stored water, delaying closure until internal stores are depleted [92,93]. Bulk pressure–volume traits influence longer-term turgor maintenance but should not be equated with the stomatal sensory mechanism because guard cells, epidermis, and mesophyll differ in osmotic regulation and mechanical constraint. In many species, conductance declines before bulk turgor loss, although moderate closure in woody angiosperms can be explained by leaf turgor when hydromechanical interactions and active regulation are included [94].

Declining outside-xylem hydraulic conductance can amplify stomatal responses by steepening the water-potential gradient required to sustain transpiration, thereby dehydrating mesophyll and epidermal tissues before extensive xylem embolism develops [95]. ABA may also modify aquaporin activity and extra-xylary water transport, while stomatal conductance determines the flux driving hydraulic decline [96]. Slow recovery of ABA concentration or hydraulic conductance can generate hysteresis, so conductance at a given VPD differs between increasing and decreasing trajectories. Apparent feedforward behaviour can thus emerge from feedback amplified by slowly reversible hormonal or hydraulic processes [54].

### 3.5. Structural and Temporal Strategies Modifying Stomatal Exposure to VPD

Stomatal size, density, pore depth, cuticular ledges, subsidiary cells, and guard-cell geometry alter maximum conductance, response speed, and the relation between turgor and aperture [56]. A structural mechanism of stomatal protection is provided through sunken, covered, or encrypted configurations, which partially isolate stomata from the external atmosphere and may reduce gas exchange [97]. In an evolutionary analysis in Proteaceae, Jordan et al. [98] demonstrated that deeply encrypted stomata have evolved repeatedly in very dry environments, suggesting a strong association with aridity.

**Figure 5:**
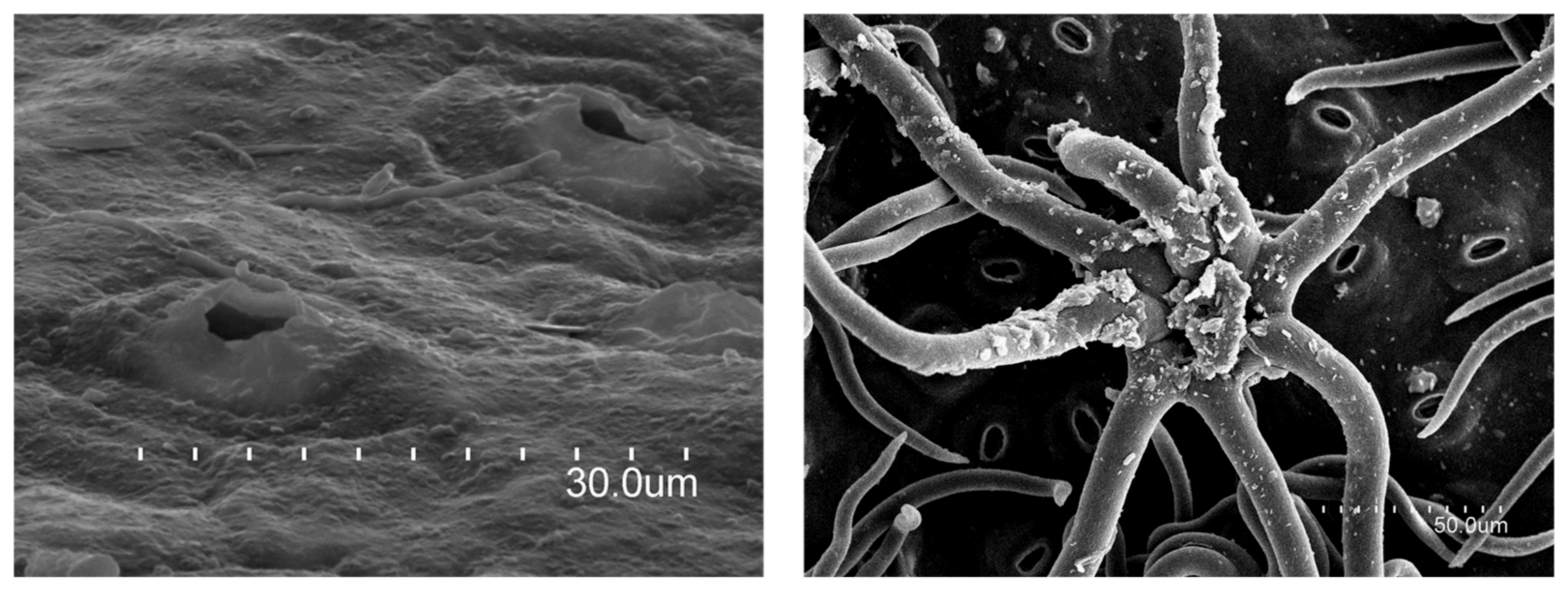
Stomatal encryption with epicuticular waxes in *Quercus coccifera* L. (**left image**) and details of the leaf abaxial trichome layer in *Quercus ilex* L. (**right image**).

Similarly, epicuticular waxes accumulated around stomatal pores can provide a plastic response to effectively reduce their functional aperture. For example, in *Quercus coccifera*, wax deposition over guard cells can drastically reduce pore area and thus strongly decrease maximum stomatal conductance under xeric conditions to optimize water-use efficiency [99] (Figure 5).

On the other hand, a dense trichome layer on the abaxial leaf surface has also been proposed to influence transpiration by modifying the leaf boundary layer and increasing diffusion resistance to water vapor loss [100] (see, e.g., Figure 5). In *Quercus* species, abaxial pubescence is more frequent in taxa inhabiting seasonally or persistently dry environments, particularly among Mediterranean evergreen oaks [101]. While this pattern is consistent with the role of trichomes in enhancing resistance to water loss, their contribution appears limited relative to stomatal and internal resistances, and empirical evidence for a major role in transpiration control remains inconsistent [102,103]. Thus, some comparative analyses indicate that trichome presence is not consistently associated with arid environments, suggesting that their function is not primarily related to water conservation [98].

The Crassulacean Acid Metabolism (CAM) represents a highly specialized strategy to mitigate the negative effects of midday VPD peaks by closing stomata [104]. CAM plants can open their stomata at night to fix CO_2_ via phosphoenolpyruvate carboxylase (PEPC), taking advantage of cooler and more humid conditions. The fixed carbon is stored as organic acids (mostly malic acid) and subsequently decarboxylated during the day, allowing internal CO_2_ refixation by Rubisco while the stomata remain closed. In this regard, CAM plants represent a specialized temporal avoidance strategy rather than the general stomatal VPD response. Nevertheless, this metabolic flexibility confers exceptionally high water-use efficiency relative to the C_3_ and C_4_ pathways and underpins the ecological success of CAM plants in arid environments [105,106]. Despite its remarkable physiological sophistication, CAM has evolved independently in multiple families, with distributions centered in arid and semi-arid ecosystems, as well as epiphytic habitats where water availability is temporarily limited [107].

Overall, stomatal responses to atmospheric dryness emerge from the integration of diffusion, tissue hydraulics, cellular mechanics, active signaling, and leaf structure. VPD increases evaporative demand, produces immediate passive changes in tissue turgor, and in many seed plants activates hydroactive regulation, with ABA playing a major but variable role. Local peristomatal water exchange may influence this integrated response, but its quantitative importance remains unresolved. Because the capacity to maintain stomatal opening ultimately depends on hydraulic supply, the following section considers long-distance water transport to transpiring surfaces.

## 4. Long-Distance Water Transport in Terrestrial Plants Under Dry Atmospheres

The development of an efficient water transport system, with water demand closely linked to transpiration during gas exchange, has been proposed as a key milestone in the evolution of terrestrial plants [108,109]. In vascular plants, the cohesion–tension mechanism efficiently drives water movement through evaporation-induced negative pressure along the plant pathway. In this model, transpiration generates negative pressure that pulls a continuous column of water through the xylem, sustained by the cohesive properties of water molecules. Although widely accepted, the cohesion–tension mechanism has been revisited [110], with modern research highlighting the role of cavitation, embolism, and additional hydraulic processes that may complement or constrain xylem transport [111]. In transpiring leaves, the ascent of xylem water is ultimately driven at evaporative interfaces within the mesophyll, where water persists as nanometric films along cell wall matrices bordering intercellular airspaces [83]. Evaporation from these surfaces induces the recession of air–water menisci confined within cell wall pores, increasing their curvature and thereby generating strong capillary (Laplace) tensions that depress local water potential [112]. This negative pressure originated at the level of mesophyll cell walls and is transmitted through the continuous liquid phase to the xylem, coupling evaporation with long-distance water transport [113].

The evolution of water-conducting systems in terrestrial plants represents a key innovation that enabled the increase in plant size and ecological diversification. Early land plants such as bryophytes developed rudimentary conducting cells (e.g., hydroids), which combined limited conductive and structural functions. Such specialized tissues comprise distinct types of water-conducting cells, typically dead at maturity but lacking lignified secondary walls, which are not homologous to the tracheary elements of vascular plants [114].

The origin of vascular plants (tracheophytes) marked a major evolutionary step with the appearance of lignified tracheids, which provided two critical advantages: (i) efficient long-distance water transport and (ii) mechanical support for vertical growth. Thus, the presence of a tracheary system in ferns could be considered a further functional step toward efficient water conduction in land plants. Ferns possess a xylem system composed primarily of tracheids with lignified secondary walls and permeable pit membranes, forming a continuous vascular network that allows long-distance water transport and resistance to cavitation [115]. Pittermann et al. [115] emphasized that (i) the structure and hydraulic function of fern tracheids reflect a balance between efficiency and safety, influenced by conduit dimensions and pit architecture, and (ii) although tracheids are homologous to those of other vascular plants, their organization and function display distinctive features related to the absence of secondary growth and the exclusive reliance on primary xylem in ferns. Tree ferns illustrate an evolutionary intermediate strategy, in which the typical fern reliance on efficient primary xylem is supplemented by structural reinforcement and enhanced lignification, enabling arborescent growth without the full development of secondary wood [109]. However, despite this considerably efficient system for conducting water, ferns are typically associated with humid environments such as cloud forests [116]. Therefore, this conduction system alone does not appear to allow survival in dry atmospheres, at least according to what is indicated by the extant genera. In fact, at a broader scale, ferns reach peak diversity and abundance in mesic habitats with stable temperatures, high rainfall, and sustained atmospheric humidity; nevertheless, a subset has evolved xeromorphic and desiccation-tolerant syndromes that facilitate persistence under water stress [117]. Notably, desiccation tolerance in ferns can be expressed with considerable plasticity, as exemplified by *Anemia caffrorum*, which produces desiccation-tolerant and desiccation-sensitive fronds in different seasons, allowing the adjustment of physiological strategies to fluctuating water availability [118].

Among gymnosperms, the tracheary system of conifers represents a major refinement in the functionality of water transport in seed plants [115]. The well-developed xylem, composed primarily of lignified tracheids arranged in continuous vascular networks, enables long-distance water transport even under very high tension [119,120]. Conifer tracheids are interconnected by specialized bordered pits with a torus–margo structure that regulates water flow and limits the spread of embolism [119], constituting the main conductive pathway and achieving a balance between hydraulic efficiency and safety [120]. By contrast, among gymnosperms, cycads exhibit a distinct vascular organization, with abundant parenchyma and tracheids forming less dense conductive tissues, often arranged in multiple vascular cylinders. Cycads retain comparatively mesomorphic features and less specialized hydraulic strategies, including lower resistance to xylem embolism [121], reflecting a stronger dependence on buffered, relatively humid environments in tropical or subtropical areas [122]. On the contrary, conifers can be found in almost all phytoclimates, from humid or very humid tropical climates [123] to arid and semi-arid ecosystems in the case of *Juniperus* species, exhibiting strong physiological adaptations to drought in terms of cavitation resistance [120]. Song et al. [124] compared 28 conifer species differing in xylem function and anatomy and found that embolism resistance was largely associated with pit structure and function, which regulate air entry between conduits. However, they did not find a clear influence of tracheid size on hydraulic conductivity or cavitation resistance measured in this set of conifers, contrary to what previous studies had suggested [125].

Further evolutionary innovation occurred in angiosperms with the development of xylem vessels, arising from tracheid-based conduction systems during the early diversification of angiosperms in the Cretaceous [126]. Their evolution involved the modification of tracheids into vessel elements with perforated end walls, enabling the formation of continuous tubular conduits [126]. In contrast to conifers, vessels are wider than tracheids, and consist of shorter cells aligned end-to-end through perforation plates, which allows a more efficient axial flow through this pathway [127]. Thus, the ability for water transport is determined by the number and size of the conduits (i.e., the fourth power of the radius), which defines the hydraulic conductivity (*K*_h_) according to the Hagen–Poiseuille law [127]. Tracheid diameter is kept within a narrow range, broadly between 10 and 30 μm, among species and families [124]. However, the variation in vessel diameter in angiosperms is extremely high, often spanning one order of magnitude [128], and reaching values up to 500 μm in some species [129]. Therefore, vessels provide a clear functional advantage over tracheids, as their greater diameter and length allow substantially higher hydraulic conductivity per unit conduit area [108], with differences in the upper limits of the variation range of two orders of magnitude compared with tracheid-based systems [127]. Higher *K*_h_ enables reduced tissue investment, greater plant size and enhanced CO_2_ uptake through sustained transpiration [108].

The xylem of angiosperms is classically grouped into two main types, diffuse-porous and ring-porous, that differ in the distribution and function of vessels within the annual growth ring, with important consequences for hydraulic architecture and seasonal water transport [127]. Diffuse-porous species are characterized by vessels of relatively uniform diameter that are distributed throughout earlywood and latewood, resulting in relatively stable hydraulic conductivity during the growing season [127]. In contrast, ring-porous species develop a distinct band of wide earlywood vessels followed by narrower latewood conduits, producing marked intra-annual variation in conductivity, with most water transport occurring in the earlywood [130]. However, this high efficiency is transient as earlywood vessels are highly vulnerable to cavitation and are typically rendered non-functional within a year due to embolism and tylosis formation, leading to a strong seasonal decline in conductivity [131]. By contrast, diffuse-porous species tend to exhibit lower maximum *K*_h_ but greater redundancy and temporal stability because conductive elements remain functional over several years [132]. This xylem type, with relative narrow vessels, predominates in most evergreen oaks [133], whereas co-occurring winter-deciduous mediterranean oaks possess a ring-porous xylem that can maintain high *K*_h_ even under periods of low secondary growth [130].

**Figure 6:**
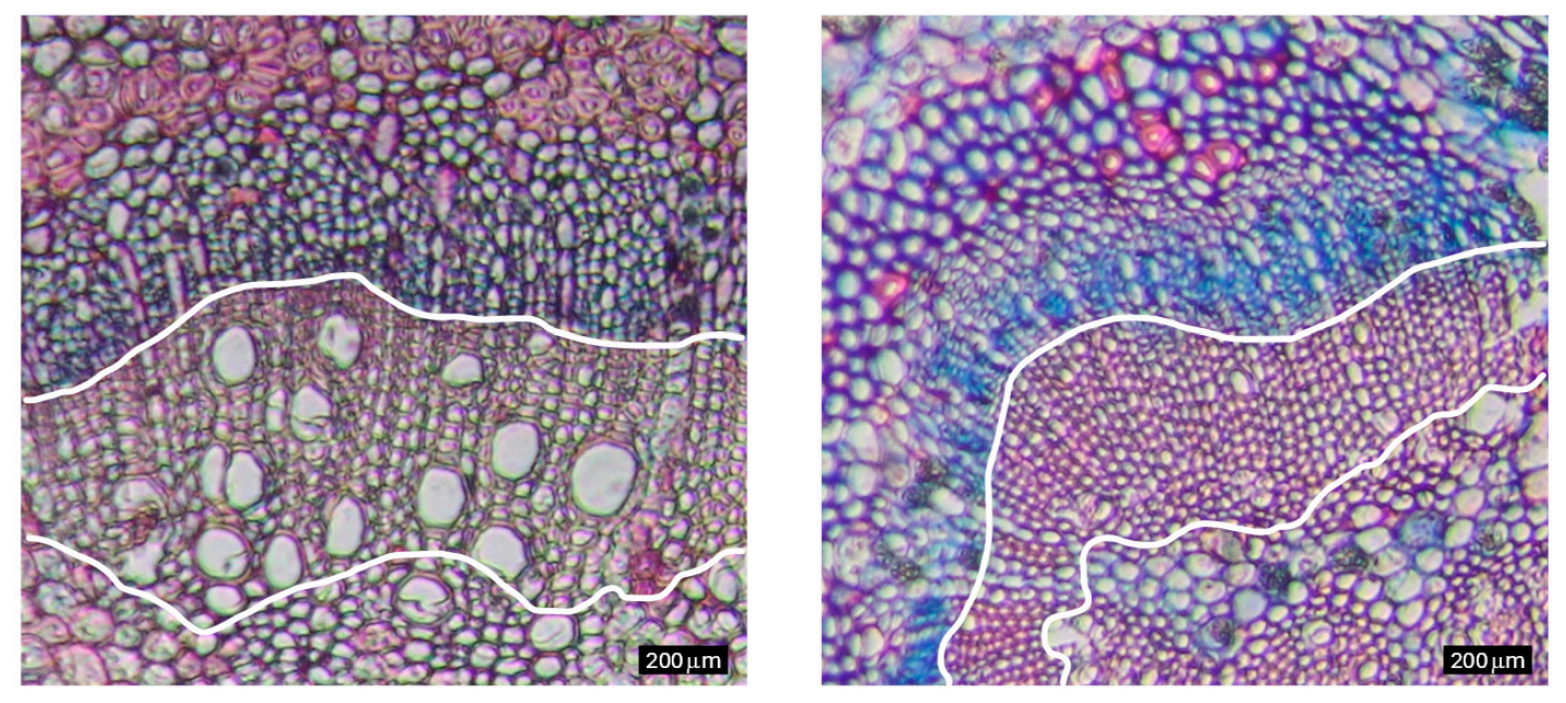
Histological cross-section of petioles showing the conduction system of two species with contrasting diameter of xylem vessels: *Quercus rubra* L. (**left image**) and *Quercus myrsinifolia* Blume (**right image**). White lines delimit xylem area.

As stated above, the efficiency of water transport through the xylem is strongly constrained by anatomical traits that are phylogenetically highly conserved (see, e.g., Figure 6). *K*_h_ adequately defines the amount of water that can pass through a given structure (root, stem, petiole) per unit time and pressure gradient. However, differences in efficiency may be influenced not only by anatomical traits (such as vessel size) but also by the amount of xylem area produced by the plant. To integrate these two sources of variation, *K*_h_ can be normalized by conductive area to obtain specific hydraulic conductivity (*K*_s_). Moreover, it should be noted that water transport throughout the plant serves to meet the water demand of transpiring leaves, so *K*_h_ can also be standardized by the leaf area supplied by the xylem, which is known as leaf-specific conductivity (LSC) [134]. The balance between water supply capacity provided by the xylem and transpiration demand imposed by the foliage can also be quantified by the Huber value (Hv), which is defined as the ratio between the conductive area and the leaf area supported, and represents a fundamental structural trait linking plant hydraulics and photosynthetic function at the whole-plant level [135].

The occurrence of atmospheres with high evaporative demand (i.e., with high values of atmospheric VPD) can increase canopy transpiration, which would induce a higher water potential drop within the plant and, consequently, an increase in the risk of hydraulic failure or catastrophic dehydration even when soil water availability is not severely reduced [7]. This situation may require a greater hydraulic sufficiency of stems to supply water to leaves, with the aim of preventing excessive declines in leaf water potential and maintaining stomata opening [136]. In this regard, Bhaskar et al. [137] demonstrated that species from Mediterranean-type climates exposed to high atmospheric VPD values displayed a shift in hydraulic traits, as a strategy to cope with the high atmospheric evaporative demand. This pattern was also assessed by Peguero-Pina et al. [138], who compared the hydraulic traits of *Abies alba* Mill. and *Abies pinsapo* Boiss., two closely related fir species that are subjected to very different environmental conditions. Thus, while *A. alba* proliferates in the humid European mountains, including the Spanish Pyrenees, *A. pinsapo* only occurs in some areas of the Mediterranean mountain ranges in Spain and Morocco, which experience intense summer drought periods and high atmospheric evaporative demand. Thus, Peguero-Pina et al. [138] reported that *A. pinsapo* showed increased *K*_s_ and LSC values in the branches when compared with *A. alba*, which can be interpreted as an adaptation to high atmospheric VPD that implied higher water transport capability to the transpiring needles. These authors also demonstrated that the higher efficiency of the xylem in *A. pinsapo* was associated with its larger tracheid diameters. However, conversely, wider xylem conduits may be more vulnerable to cavitation [139], which ultimately can lead to hydraulic failure and plant mortality [140]. The possible existence of a trade-off between xylem efficiency and vulnerability to cavitation has been subject of investigation since the very early studies in plant hydraulic architecture [141]. Thus, Martínez-Vilalta et al. [142] showed empirical evidence for such a trade-off between efficiency (*K*_s_) and safety when comparing several Mediterranean woody species, mainly angiosperms. However, the development of tracheids with higher protection against cavitation caused by water stress seems to be uncoupled from conduit size and conducting efficiency [128].

In addition to increasing the number and/or size of the conduits, another effective mechanism for increasing xylem efficiency to supply water to the leaves is the reduction of transpiring area when VPD is very high. In this regard, Peguero-Pina et al. [143] compared several provenances of *Quercus ilex* and found a clear improvement in the LSC for those occurring in more xeric sites, which was explained by a reduction in leaf size and whole leaf area per shoot, rather than by an increase in sapwood area or the existence of a more efficient xylem (e.g., through an increase in *K*_s_). Similarly, Peguero-Pina et al. [144] found that the transpiring area per shoot of the Mediterranean oak *Quercus faginea* Lam. was ca. 6 times lower than *Quercus robur* L., a species only present in temperate climates in the absence of atmospheric dryness. As a result, *Q. faginea* increased its ability to supply water to transpiring leaves at shoot level, reaching LSC values very similar to those measured for *Q. robur* despite the sharp difference found in *K*_h_ between both species (ca. 7 times). This strategy could contribute to maintaining stomata opening under the higher atmospheric VPD values experienced by *Q. faginea* throughout the vegetative period. Regarding gymnosperms, several studies have also found a reduction in the amount of leaf area in relation to the conductive xylem area with increasing atmospheric evaporative demand in several conifers [145,146].

## 5. Drought-Avoidance Strategies Under Dry Atmospheres

Drought avoidance refers to strategies that limit tissue dehydration, in contrast to drought tolerance, which enables cells and tissues to remain functional at lower water content. Under atmospheric dryness, avoidance strategies are often described along a continuum between conservative “water savers”, which regulate strictly water loss, and acquisitive “water spenders”, which maintain transpiration while water supply is sufficient [147]. However, this terminology should be presented as context-dependent phenotypic response and not be interpreted as a fixed species classification. Recent syntheses show that water-use behavior is an emergent property of plant hydraulic traits, rooting access, and environmental forcing, and that the apparent position of a species along the conservative-acquisitive continuum can shift with soil water availability and VPD [8,148]. This distinction is particularly important for the present review because responses to rising VPD operate predominantly at sub-daily to daily scales, whereas responses to progressive soil drying integrate belowground limitations over longer periods; consequently, a genotype or species that behaves conservatively under high VPD is not necessarily conservative during seasonal soil drought [149].

**Figure 7:**
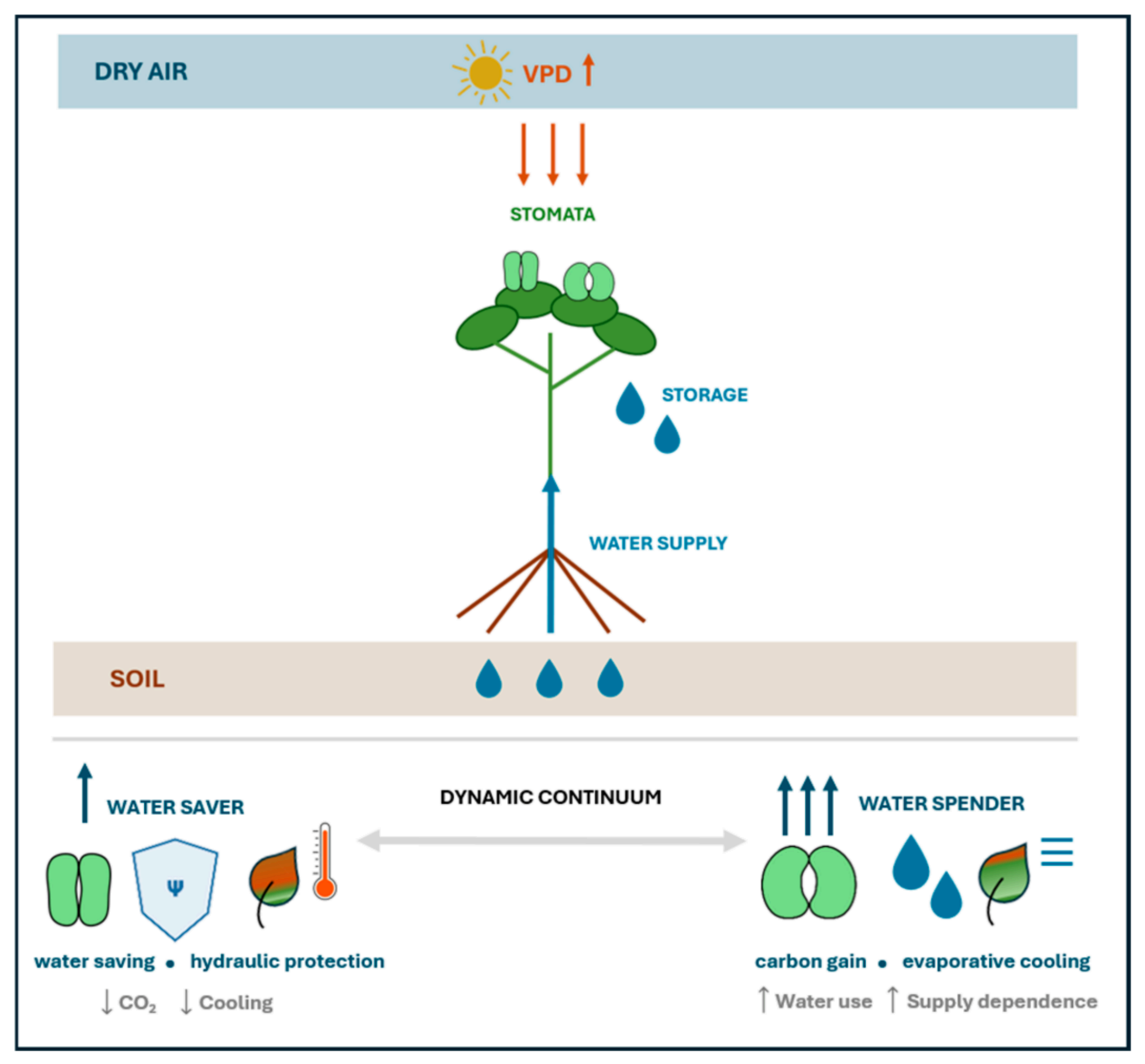
**Dynamic whole-plant coordination of drought-avoidance strategies under high atmospheric vapor pressure deficit (VPD).** Increasing VPD enhances atmospheric evaporative demand, whereas the capacity to sustain transpiration depends on the balance between soil water availability, root water access, hydraulic supply and internal water storage. At the conservative end of the continuum, water-saving responses involve earlier stomatal restriction, reducing transpiration and hydraulic exposure but simultaneously limiting CO_2_ assimilation and evaporative cooling. At the acquisitive end, water-spending responses maintain stomatal opening, carbon assimilation and leaf cooling for longer, at the cost of greater water consumption and stronger dependence on water supply. These responses should not be regarded as fixed species categories, but as context-dependent positions along a dynamic continuum that shifts with VPD, soil water availability and whole-plant hydraulic status. **Symbol legend.** Red downward arrows indicate increasing atmospheric evaporative demand associated with high VPD. The central plant represents whole-plant coordination between stomatal regulation, root water uptake and hydraulic transport. Blue drops represent available water pools, either in the soil or in internal plant storage. Upward arrows indicate relative transpiration and water loss, with fewer arrows representing stronger water conservation and more arrows representing greater water use. The Ψ shield represents protection of plant water status and hydraulic integrity. Leaf symbols represent leaf-level carbon assimilation and associated physiological activity, whereas the thermometer indicates leaf thermal status and evaporative cooling. On the water-spending side, the combination of high transpiration, water-pool symbols and the leaf/cooling icon represent sustained gas exchange and evaporative cooling supported by greater water supply.

At the water-saving end of this continuum, rapid restriction of transpiration reduces the decline in leaf water potential and delays depletion of internal water pools, thereby increasing drought avoidance at the cost of lower instantaneous carbon gain (Figure 7). The contrasting behaviour of *Q. coccifera* and *Pistacia lentiscus* L. exemplifies this functional axis in Mediterranean shrubs [150]. *Q. coccifera* shows a highly conservative water-saving strategy, characterized by intrinsically low stomatal conductance even under well-watered soil conditions, which strongly limits transpiration in response to dry atmospheres, together with low hydraulic conductivity. In contrast, *P. lentiscus* adopts a water-spending strategy in which higher hydraulic conductivity enables a more efficient supply of water to the leaves, allowing sustained stomatal opening and higher transpiration rates even under conditions of elevated atmospheric demand. The tight stomatal regulation of *Q. coccifera* allows this species to buffer increases in VPD, maintaining relatively stable leaf water potentials and wide hydraulic safety margins despite high atmospheric demand [147,150]. Conversely, the higher hydraulic conductance of *P. lentiscus* supports steeper hydraulic–photosynthetic relationships, enabling greater gas exchange capacity under dry atmospheric conditions until hydraulic constraints become limiting [151]. However, this strategy is more dependent on access to reliable water sources; consequently, *Q. coccifera* can dominate large areas of the landscape, whereas *P. lentiscus* tends to be restricted to specific microsites where water availability is higher or more sustained.

Although the water-saver/water-spender dichotomy appears to be a general functional axis structuring Mediterranean plant communities [152], these labels are more appropriately applied to the dynamic response phenotype rather than treated as immutable species attributes. An important consequence of stomatal regulation under dry and hot atmospheres is that water conservation and thermal regulation may become antagonistic. Restricting transpiration reduces hydraulic exposure but also suppresses evaporative cooling, whereas maintaining some water loss can protect leaves from overheating [153]. Field measurements in *Quercus petraea* (Matt.) Liebl., *Q. ilex*, and *Q. coccifera* showed that canopy temperature could exceed air temperature by up to approximately 10 °C when net CO_2_ assimilation approached zero or became negative. Nevertheless, the two Mediterranean oaks (*Q. ilex* and *Q. coccifera*) still maintained positive stomatal conductance under null or even negative carbon fixation, a stomatal decoupling that may contribute to evaporative cooling during extreme heat [154]. Therefore, under hot–dry conditions, the relevant trade-off is not simply carbon gain versus water saving, but a three-way balance among hydraulic safety, carbon assimilation, and avoidance of thermal damage. Consequently, depending on the prevailing environmental conditions, typical water-savers may show suboptimal stomatal regulation as a heat-avoidance strategy.

The conservative-acquisitive continuum should also be distinguished from iso-/anisohydric behaviour and from drought survival per se. In temperate trees, stomatal stringency was related to the time required to reach stomatal closure, but not to the subsequent time to critical hydraulic failure, which depended more directly on xylem safety [155]. Moreover, recent field observations in mature trees indicate that stomata may close much earlier during dehydration than assumed by conventional iso-/anisohydric metrics, questioning the use of hydroscape-derived classifications as direct proxies for stomatal regulation across the full drought trajectory [156]. These results support using “water saver” and “water spender” as heuristic endpoints of a context-dependent continuum rather than as fixed functional types.

At the water-spending end, sustained gas exchange is possible only when transpiration demand is coordinated with water access and hydraulic supply (Figure 7). A recent survey of 62 native woody species across 10 Mediterranean sites showed that above- and below-ground water-use traits are strongly coordinated: larger and taller species accessed a greater proportion of deep soil water and displayed more conservative leaf-level water use, whereas smaller species relied predominantly on shallow water and showed more profligate water-spending behaviour [157]. These results extend the saver–spender concept from a leaf-level description to a whole-plant eco-hydrological syndrome and indicate that drought and heat strengthen trade-offs among water uptake depth, stomatal regulation, and biomass allocation. In this view, no single stomatal or hydraulic trait is sufficient to define drought-avoidance strategy.

Internal water storage provides an additional layer of plasticity within this continuum. Recent work comparing *Pinus sylvestris* and *Quercus faginea* showed that the water-saving pine rapidly closed stomata and limited stem dehydration and water uptake, whereas the more water-spender oak maintained transpiration by drawing on elastic stem water storage and displayed different water-replacement dynamics after rewatering [158]. Thus, drought avoidance depends not only on stomatal sensitivity but also on how water fluxes and internal storage are coordinated through the whole plant. Modelling constrained by field measurements in *Fagus sylvatica* and *Picea abies* showed that hydraulic capacitance can buffer leaf water potential and support transpiration, but that its effect depends on stomatal regulation: high capacitance enhanced transpiration under strict water-potential regulation, whereas under a more flexible strategy it could induce earlier stomatal down-regulation under wet-soil conditions [159]. Because leaf-level capacitance and hydraulic conductance are considered in Section 3.4, the key point here is their whole-plant consequence: similar stomatal responses can produce different drought-avoidance outcomes depending on accessible internal water storage and the temporal coupling between hydraulic supply and atmospheric demand.

The limitations of an excessively conservative response are illustrated by silver fir (*Abies alba* Mill.) near its south-western distribution limit. This species is highly sensitive to atmospheric dryness, with strong reductions in stomatal conductance and photosynthesis as VPD increases even before soil moisture becomes critically limiting [160,161], a pattern also observed in the Mediterranean evergreen oak *Q. ilex* [162]. Long-lasting restrictions on gas exchange can reduce carbon supply and growth, but current mortality frameworks no longer support treating carbon starvation as an isolated alternative to hydraulic failure [163]. Instead, carbon limitation, water depletion, hydraulic dysfunction, and biotic interactions are increasingly understood as interdependent processes whose progression can be accelerated by rising VPD [163], consistent with field evidence linking hydraulic dysfunction with reduced growth, low carbohydrate reserves, and increased pest infestation following severe drought [164]. Thus, a water-saving strategy can be effective for short-term drought avoidance while still carrying substantial long-term costs when atmospheric dryness becomes chronic.

Overall, drought avoidance under dry atmospheres emerges from the coordination of stomatal regulation, water access, internal storage, and thermal regulation rather than from a single optimal degree of water saving (Figure 7). Conservative strategies reduce short-term hydraulic exposure but constrain carbon gain and evaporative cooling, whereas acquisitive strategies sustain photosynthesis and cooling at the expense of faster water use and greater dependence on water access. The most informative framework is therefore a dynamic saver–spender continuum whose position changes with VPD, soil water availability, and whole-plant hydraulic context [8,157]. Within this continuum, the coordination between stomatal regulation and photosynthesis could be influenced by the expected increases in atmospheric CO_2_ concentration, which may reduce the magnitude of some negative responses by enhancing water-use efficiency and maintaining photosynthesis. However, rising air temperatures would promote the sustain of stomatal decoupling to maintain evaporative leaf cooling [165]. Further research should consider the three-way balance among hydraulic safety, carbon gain, and avoidance of damaging leaf temperatures in the context of increasing air temperatures associated with global warming. This would be carried out considering a wide phylogenetic spectrum, with a multiple trait integration from organs to whole plants and models.

## 6. Conclusions

Atmospheric dryness, commonly quantified as VPD, represents a major environmental constraint that directly influences plant water relations across the entire soil–plant–atmosphere continuum (SPAC). This review highlights that high VPD is not merely a driver of transpiration, but a central factor shaping plant structure, physiology, and survival, even when soil water availability is not limiting. Plant responses to increasing evaporative demand emerge from the interaction among protective barriers, stomatal regulation, hydraulic transport systems, and whole-plant drought-avoidance strategies.

The cuticle is one of the most critical innovations enabling land colonization and plant survival in terrestrial habitats, constituting the first line of defense against atmospheric desiccation. There is increasing evidence indicating that its effectiveness depends not only on thickness but also on its chemical composition, thermal stability, structural heterogeneity, and environmental responsiveness. Residual water loss through the cuticle can become critically important once stomata close, particularly under high-VPD conditions where increases in cuticular conductance may accelerate tissue dehydration.

Stomatal responses to VPD arise from the integration of tissue hydraulics, cellular mechanics, and active biochemical regulation. Current evidence strongly supports hydroactive regulation, frequently involving ABA signaling, as a key mechanism underlying stomatal closure in seed plants. The classical peristomatal transpiration hypothesis is better interpreted as a potential modifier of the amplitude or kinetics of hydroactive feedback than as a universal explanation of VPD responses.

The capacity to sustain gas exchange under high atmospheric demand ultimately depends on hydraulic supply. The evolution of increasingly efficient vascular systems, from tracheid-based pathways to vessel-bearing xylem, enabled enhanced water transport and greater photosynthetic performance, but also introduced trade-offs between hydraulic efficiency and vulnerability to dysfunction. Consequently, plant responses to high VPD cannot be understood without considering the coordination between stomatal regulation, hydraulic architecture, and the balance between water transport capacity and transpirational demand.

At the whole-plant scale, drought avoidance should be viewed as a dynamic continuum between water-saving and water-spending strategies. Conservative strategies reduce hydraulic risk but may limit carbon gain and evaporative cooling, whereas acquisitive strategies sustain gas exchange and thermal regulation at the expense of greater dependence on water availability. These two strategies are complementary physiological solutions that jointly coordinate stomatal regulation, xylem hydraulic traits, and carbon reserve dynamics to cope with atmospheric water deficits. Recognizing this diversity of drought-avoidance mechanisms, rather than assuming a single optimal strategy, is essential for anticipating species-specific vulnerability and community-level resilience under increasingly frequent and intense drought events.

Future research should move beyond the analysis of isolated traits and adopt an integrated perspective linking atmospheric evaporative demand, protective barriers, carbon metabolism, plant hydraulics, and whole-plant drought-avoidance strategies. Improving our understanding of how plants coordinate these processes will be essential for predicting plant performance, ecosystem functioning, and species vulnerability under increasingly dry atmospheric conditions associated with ongoing climate change.

## Figures and Tables

**Figure A1:**
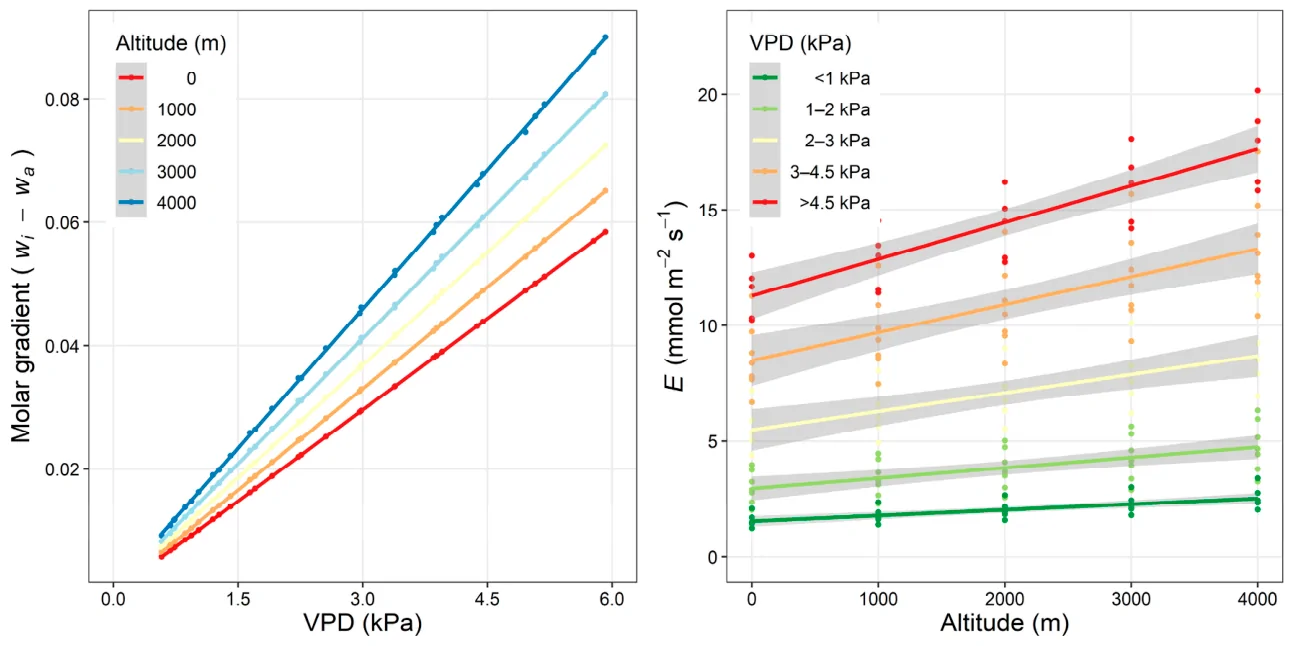
**Effect of changing atmospheric pressure with altitude. Left panel**: Association between leaf-to-air water pressure deficit (VPD) and the water-vapor mole-fraction gradient (*w_i_ − w_a_*) for different altitudes. **Right panel**: Implication of changing *w_i_ − w_a_* with altitude for the response of transpiration (*E*) to VPD. Calculations made on different combinations of temperature (7.5–50 °C) to achieve a similar range of VPD within three levels of relative humidity (30%, 40%, 60%). *E* was determined using the simplified formula shown in Equation (1), assuming *T_leaf_* = *T_air_*, saturating atmosphere inside the leaf, and a constant *g_tot_* = 200 mmol m^−2^ s^−1^.

## Data Availability

Data are available on reasonable request.

## Appendix A. Beyond VPD: Implications of Pressure Changes on Transpiration Demand

As mentioned above, transpiration rate is more directly related to the leaf-to-air water-vapor mole-fraction gradient (*w_i_ − w_a_*) than to the corresponding water-potential difference. Considering ideal-gas assumptions and similar total pressure inside and outside the leaf, this molar gradient can be approximated as *w_i_ − w_a_* ≈ (*e_i_* − *e_a_*)/*P_atm_*, and transpiration as *E* ≈ *g_tot_*×(*e_i_* − *e_a_*)/*P_atm_*, where *e_i_* − *e_a_* stands for the vapor pressure deficit (VPD) from the leaf to the atmosphere, *P_atm_* is atmospheric pressure and *g_tot_* is total conductance to water vapor. This formulation explains why VPD is a better proxy for transpiration demand than the water-potential gradient, but also evidences that VPD values are only comparable within a similar range of *P_atm_*. One major source of variation for *P_atm_* is altitude, and its effect assuming a constant temperature can be estimated as follows [5]:

(A1) $$P_{z}\approx P_{0}\times e^{-\frac{\mathrm{Mgz}}{\mathrm{RT}}}$$

where *P_z_* is the atmospheric pressure at height *z*, *P*_0_ is the reference pressure at sea level (101.325 kPa), *M* is the molar mass of air (0.028964 kg mol^−1^), *g* is the acceleration due to gravity (9.8 m s^−2^), *R* is the universal gas constant (8.314 J K^−1^ mol^−1^) and *T* is absolute temperature (K). Within the typical pressure variation due to meteorological conditions, or along a small altitude range (ca. 500 m), the effect of pressure is negligible when compared to the variability in other meteorological variables, such as temperature and relative humidity [6]. However, if we aim to compare plant responses to atmospheric dryness along an altitudinal gradient, or at a global scale, the effect of altitude becomes relevant. In Figure A1 we exemplify the association between leaf-to-air VPD and the molar gradient across different altitudes, and the implications in terms of transpiration. From these calculations, it becomes evident that the association between VPD and transpiration and the subsequent stomatal response may differ considerably along a wide altitudinal range.
